# Nanoparticle shapes by using Wulff constructions and first-principles calculations

**DOI:** 10.3762/bjnano.6.35

**Published:** 2015-02-03

**Authors:** Georgios D Barmparis, Zbigniew Lodziana, Nuria Lopez, Ioannis N Remediakis

**Affiliations:** 1 Department of Materials Science and Technology, University of Crete, Heraklion, 71003, Greece; 2 Department of Physics and Astronomy, Vanderbilt University, Nashville, TN, 37235, USA; 3 INP, Polish Acad Sci, ul. Radzikowskiego 152, PL-31342 Krakow, Poland; 4 Institute of Chemical Research of Catalonia, ICIQ, Av. Paisos Catalans 16, Tarragona 43007, Spain

**Keywords:** density functional theory (DFT), hydrogen storage, multi-scale simulations, nanoparticles, surface energies, surfactants, Wulff construction

## Abstract

**Background:** The majority of complex and advanced materials contain nanoparticles. The properties of these materials depend crucially on the size and shape of these nanoparticles. Wulff construction offers a simple method of predicting the equilibrium shape of nanoparticles given the surface energies of the material.

**Results:** We review the mathematical formulation and the main applications of Wulff construction during the last two decades. We then focus to three recent extensions: active sites of metal nanoparticles for heterogeneous catalysis, ligand-protected nanoparticles generated as colloidal suspensions and nanoparticles of complex metal hydrides for hydrogen storage.

**Conclusion:** Wulff construction, in particular when linked to first-principles calculations, is a powerful tool for the analysis and prediction of the shapes of nanoparticles and tailor the properties of shape-inducing species.

## Introduction

The functionality of nanoparticles in modern materials is intimately linked to their structure in terms of size and shape [1]. Structures at the nanoscale have found myriads of applications out of the traditional field of heterogeneous catalysis and more are discovered almost on a daily basis. Nowadays, nanoparticles can be found in sensors, especially with biomedical interest, as agents to induce the death of cancer cells, as drug delivery vehicles, in emerging energy technologies, either in harvesting or for storage, as additives for fuels, in optics, and as part of smart fluids, just to name a few applications. The “plenty of room at the bottom” described by Feynman, is thus being filled with technological uses [2]. Indeed, several road-maps have been established to improve our knowledge and control for such powerful technologies. It is well-known that, for nanotechnology to be successful, a higher degree of control over the synthesis at the nanoscale, better characterization techniques and simulation methods are required. The main aim of simulations is to provide guiding tools for the tailored synthesis of desired architectures. Concerning nanoparticles, a high degree in nanoparticle size control was achieved very early. This is demonstrated by the famous Lycurgus cup, that changes color due to directional light scattering combined with adsorption by the nanoparticle plasmon resonances, or the colorful windows in the facades of medieval cathedrals. Therefore, the real challenge we are facing today is to be able to control the shapes of nanoparticles [3]. The shape of nanoparticles can have a big impact on their properties, not only in catalysis in which the number of active sites is clearly shape-dependent [4], but also in other applications such as optics [5–6].

Theoretical simulations based on the Wulff construction hold the key to understand the shapes of nanoparticles. The Wulff construction revolutionized geology and crystallography especially at the beginning of the twentieth century, as it provided a systematic way to classify characteristic shapes and habits found in mineral crystals. Near the end of the twentieth century, Wulff construction was re-discovered in materials science and was used to characterize shapes of nanoparticles. Today, multi-scale simulations that use Wulff constructions based on first-principles quantum-mechanical calculations of surface energies are routinely employed to explain experimental findings and lead to the design of better materials with tailored properties.

In the following, we review the concept of Wulff construction, present its mathematical formulation and limitations, and review modern uses in the analysis and the prediction of findings in microscopy experiments. We then present three recent extensions to this methodology: (a) the atomistic Wulff construction that allows for the detailed analysis of nanoparticles at the atomic level; (b) the inclusion of surfactants that gives rise to nanoparticles with shapes that have a lower symmetry than the bulk form of the same material and (c) Wulff constructions for complex materials with many metastable crystal structures.

## Review

### Wulff construction: definition and basic properties

In his famous paper of 1874 ”On the Equilibrium of Heterogeneous Substances”, J. Willard Gibbs concluded that a given quantity of matter will attain a shape such that the total surface energy is minimal [7]. For perfect crystalline solids, atomic planes are members of a countable set characterized by integer Miller indexes (*hkl*). The shape of a crystalline solid will therefore be a polyhedron for which only faces parallel to (*hkl*) planes are allowed. It is only reasonable to assume that faces with a relatively low surface energy will dominate the equilibrium shape.

Several decades later, mineralogist Georg Wulff suggested [8] that the polyhedron that corresponds to the lowest surface energy of a crystalline substance can be constructed in the following way (the so-called Wulff construction): One chooses a constant *c*, and a Cartesian set of axes. Starting from the origin, *O*, one draws a plane that is normal to the [*hkl*] vector and has a distance *d_hkl_* = *c*·γ*_hkl_* from *O*. The quantity γ*_hkl_* is the energy required to create a surface of unit area normal to the [*hkl*] vector, and is the analogous of the surface tension for liquids. This process is repeated for all sets of Miller indexes, (*hkl*). The space that lies inside all these planes defines the equilibrium shape for this material. The first proof of the Wulff theorem came in 1943 by von Laue [9]; for more details, see [10–11]. More general proofs of Wulff’s theorem have appeared in the recent years [12], and it is still a subject of on-going research in applied mathematics [13]. For instance, the Wulff–Kaishew theorem is a generalization that considers lateral strain [14].

When the material under study is at equilibrium with another gas- or liquid-phase material, the interface tension, 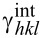, is used in the Wulff construction instead of the surface tension, γ*_hkl_*. The two are connected by a simple formula that involves the surface coverage, θ, the adsorption energy, *E*_ads_, and the area per surface atom, *A*_at_ [15]:

[1]
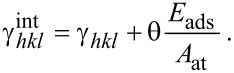


Interactions between adsorbates are implicitly taken into account in Equation 1, as these interactions will affect the values of both *E*_ads_ and θ.

**Figure 1:**
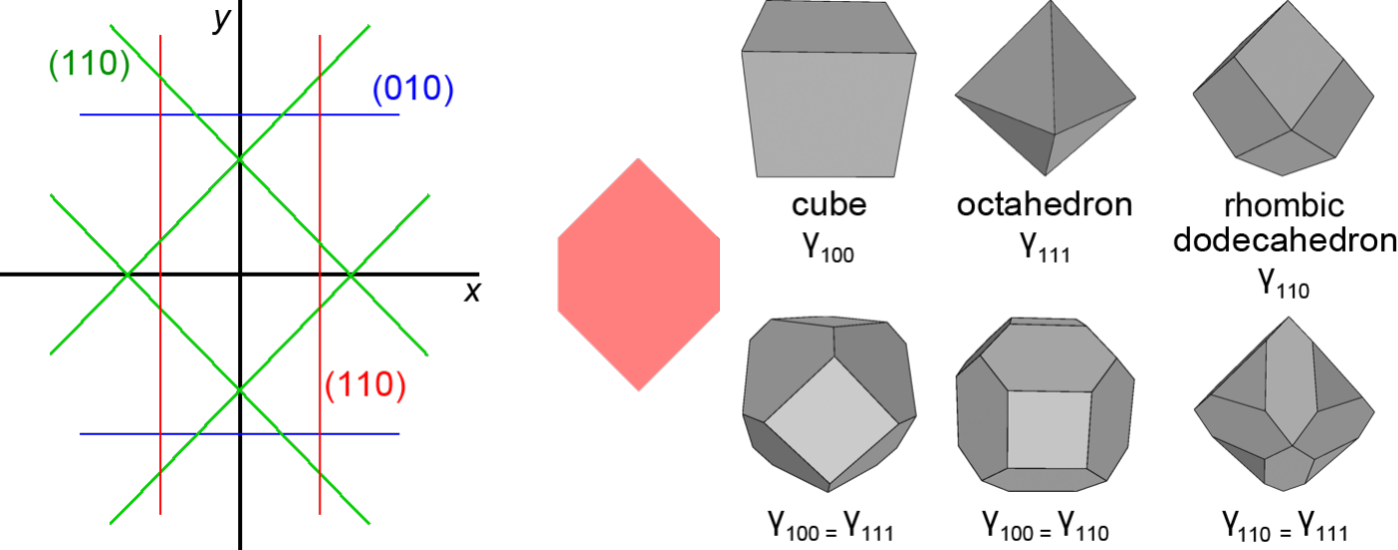
Left: Example of Wulff construction for orthorhombic material. *xy* plane is parallel to the (001) plane of the material. Center: The cross-section of the resulting equilibrium shape. Right: Some common Wulff constructions for materials with full cubic symmetry (*O_h_* or 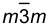), such as fcc metals. The surface energies that have been used in the Wulff construction are shown below each shape.

An example of Wulff construction is shown in Figure 1 for a hypothetical orthorhombic material with 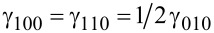. All other (*hkl*) planes have much higher surface energies. The resulting equilibrium shape is a rod-like prism. Interestingly, the cross-section of the crystal is an (almost regular) hexagon, although the material does not posses hexagonal symmetry. Similar pseudo-hexagonal shapes are often found in minerals of orthorhombic crystals, such as aragonite CaCO_3_ [16].

The Wulff construction results in a polyhedron that:

1. depends only on ratios between surface tensions and not on their absolute values;
2. contains few faces with high Miller indexes as in most cases, they have a much higher surface tension than low-index faces; (if a high-index surface and a low-index surface have equal surface tensions, the low-index will have a greater area as the high-index face will be steeper and will be hidden in the Wulff construction);
3. does not take into account edge- and vertex-energies; and
4. bears the same symmetry (belonging to the same point group) as the bulk material.

Point 2 of the aforementioned list allows for a truncation of the set of (*hkl*) planes for small values of *h*, *k* and *l*. In most cases, the essential features of the shape can be found by considering only planes with indexes of zeros and ones. An exception might be nanoparticles with strong interactions with their environment. In that case, high-index surfaces that contain steps and kinks offer better binding of adsorbates which will lower their surface energy (see Equation 1). However, due to the fact given in brackets in point 2, the equilibrium shape rarely contains faces with indexes higher than three [17].

Point 4 is correct at the thermodynamic limit for very large nanoparticles. Smaller nanoparticles that have Wulff shapes could in principle belong to a subgroup of the original point group, as the center of the nanoparticle might not coincide with the symmetry center of the crystal structure. For Au nanoparticles, for example, two different classes of nanoparticles can be found depending on whether the center of the nanoparticle is at an fcc lattice point or at the octahedral site of the structure. However, these two types become indistinguishable for nanoparticles larger than a few angstroms [15]. Some characteristic equilibrium shapes that are often found in materials with cubic symmetry are shown in the right panel of Figure 1.

Symmetry, through point 4, can be used to rule out shapes of larger nanoparticles that are clearly out of equilibrium. However, it was recently found that when complex ligands are attached to their surfaces, nanoparticles may have equilibrium shapes that do not belong in the same point group as the material (see section about ligands below).

### Wulff construction in the modern era: shapes of nanoparticles

In the past, equilibrium shapes were only associated with minerals and gemstones. Superb chemical stability and equilibration over millions of years under extreme pressure and temperature seemed to be the necessary (but often not sufficient) conditions for a piece of matter to be in its equilibrium shape. In the last two decades, however, it has been realized that due to their small sizes, nanoparticles can reach equilibrium within seconds or less. Moreover, the equilibrium shapes of nanoparticles are the same as those of gemstones with the same chemical composition. For example, the octahedral shape characteristic of natural diamonds is also observed for nanoparticles that consist entirely of C atoms in the diamond crystal structure [18].

The Wulff construction offers a simple and rigorous way to describe nanoparticle shapes without the need to use complex mathematical language. The shapes can be described by two or three parameters (the surface energies of some (*hkl*) faces and the point group of the material). These parameters can be plugged in to a Wulff construction code to reproduce the shape of the nanoparticle. Popular examples of free Wulff construction implementations are Wulffman [19] and VESTA [20].

Among the first uses of the Wulff construction for the characterization of nanoparticles are experiments by H. Topsøe and co-workers for Cu-based catalysts [21–22], and the theory of Müller and Kern for epitaxially strained semiconductor quantum dots [23]. Since then, Wulff shapes have been observed in a variety of microscopy experiments with nanoparticles. Some characteristic examples include Ru [24], Pt [25], Au [26–30], Ni [31] and Si [32] nanoparticles.

The increase of computational power and the emergence of smart codes for the electronic structure of materials allowed for calculations of interface tensions from first principles. These data were often used in Wulff constructions for the prediction of the shape of nanoparticles in a variety of environments. Some characteristic examples include supported Au [33–34], diamond [35], TiO_2_ [36], Si in amorphous SiO_2_ [37], diamond in amorphous C [38], Rh and Pd under oxidizing conditions [39], Cu in N gas [40], Au under oxidizing conditions [41], noble metals with an environment [42], complex metal hydrides [43], iron carbides [44] and dawsonites [45–46], just to name a few.

### Atomistic Wulff construction

The atomistic Wulff construction emerged through the field of heterogeneous catalysis, in which the shape of transition metal nanoparticles is a key factor of their functionality. Atomistic models for an equilibrium-shaped Ru nanoparticle were part of a long-term project to model an industrial ammonia-synthesis catalyst from first principles [47–49]. Density functional calculations were used to obtain accurate surface energies for several faces of hcp Ru. These values were used in a standard Wulff-construction software to create the polyhedron that corresponded to the equilibrium shape. In the last step, this polyhedron was filled with atoms in order to create a model of a cluster at thermodynamic equilibrium. This model was then used to calculate various atomistic properties of Ru nanoparticles that were measured experimentally, such as the number of moles of B-type step atoms per gram of catalyst.

Nanoparticles that are relevant to catalysis have diameters of the order of 5 nm and contain of the order of 5000 transition metal atoms. Direct simulation of such systems by using quantum-mechanical techniques is difficult at present. Modern DFT codes that can tackle several thousands of atoms are presently limited to atoms with few valence electrons, such as simple metals or semiconductors [50–52]. Late transition metals are typically simulated by using advanced force-fields or other approaches [53–55]. The advantage of the atomistic Wulff construction is that one avoids being trapped at one of the metastable structures of the nanoparticle as they do not need to span the full configurational space of the problem.

The turnover frequency (TOF) of a catalyst is proportional to the density of active sites (usually expressed as μmol of active sites per gram of catalyst). Microscopy and/or first-principles simulations are typically used to identify what the active sites for each catalytic reaction are. For example, B-type step atoms are the only active sites for ammonia synthesis on Ru-based catalysts [56]. Atomistic Wulff constructions can be then used to calculate the number of such atoms in a nanoparticle of a given diameter [47–49]. This procedure was recently used to account for the nanoparticle size-dependent TOF of a Ag catalyst in the selective semi-hydrogenation of alkyne–alkene mixtures [57].

Another advantage of the atomistic Wulff construction is that it enables the study of the equilibrium shape of the nanoparticle as a function of its size. The Wulff polyhedron is the equilibrium shape of the nanoparticle at the thermodynamic limit of large particles. At the nanoscale, some faces of the Wulff polyhedron might be so small that they cannot accommodate a single atom, let alone a unit cell of the (*hkl*) plane they represent. This leads to shapes for small nanoparticles that are significantly simpler than the Wulff polyhedron. For example, Au nanoparticles with diameter less than 16 nm contain only (111) and (100) surfaces, although the Wulff shape contains (111), (332), (100), (211), and (322) [15].

**Figure 2:**
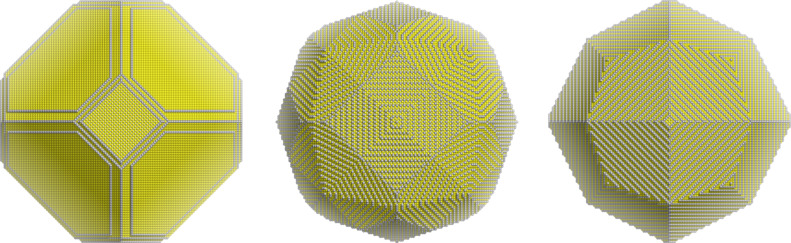
Atomistic Wulff constructions for Au nanoparticles using surface energies published in Refs. [15] and [17]. Atoms that have coordination numbers less than nine are colored in gray. Left: in vacuum or inert environment (28 nm in diameter, 539500 atoms, 2980 of which are step-edge atoms; (111) faces occupy 62% of the total area). Center: In low-pressure CO gas (27 nm in diameter, 533600 atoms, 7610 of which are step-edge atoms; (321) faces occupy 61% of the total area). Right: With adsorbed SCH_3_ radicals (29 nm in diameter, 502900 atoms, 10600 of which are step-edge atoms; (211) faces occupy almost 100% of the total area).

An example of atomistic Wulff construction for relatively large Au nanoparticles is shown in Figure 2. Upon exposure to CO or CH_3_S radical gas, the nanoparticle changes towards more spherical shapes, in accordance with experiments [58].

### Nanoparticles with surfactants

Metal-only nanoparticles or metal-adsorbate interactions have been the leading force that has helped the evolution of the presented methodology for the shape of nanocrystals. However, recent developments to design colloidal suspensions of nanoparticles with interesting physical and chemical properties have pushed forward the simulation frontier. Indeed, the synthesis of nanoparticles through wet chemistry methods is usually quite complex. When nanoparticles are to be employed in optics or as sensors, many formulations employ the seed-growth methodologies. First a nucleus (1–2 Å diameter) is formed by the fast reduction of a metal salt and in a subsequent step these nanoparticles are placed in a second solution in which they are allowed to grow. The second solution contains an important amount of surfactants, in some cases secondary metals and other shape inducing agents, typically halides. Depending on the mixture of surfactants, secondary ions and anions nanoparticles with different shapes can be observed [3]. From the point of view of modeling, the growth of these nanoparticles is difficult, but relevant information can still be extracted. For instance, the preferential adsorption of halides to particular facets reduces the surface energy of those facets and typically they are represented more in the equilibrium state of the particle. This has been observed for the appearance of nanocubes of Ag grown on Au seeds in the presence of chlorine [59].

**Figure 3:**
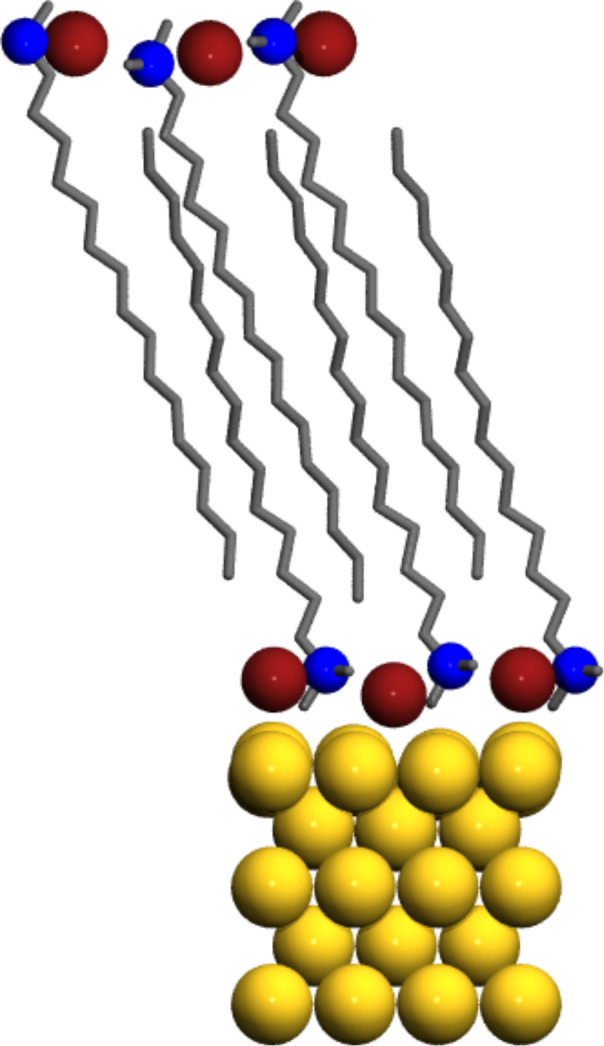
Schematic representation of the adsorption of a surfactant on a gold surface. Spheres represent gold (yellow), silver (grey) and bromine (red) and the tails of the surfactant are represented by sticks.

In some cases a symmetry breakdown (i.e., that the nanoparticle does not belong strictly to the same point group as the bulk) of the metal particle is desired, as nanoparticles with spikes have enhanced spectroscopic features. According to the Wulff rules this is not possible (see above the constraint in point 4). However, experimentally it has been found that objects such as nanorods can be systematically produced with an adequate selection of the solution growth. In that case the symmetry breaking appears very early in the formation of the seed as then some of the facets (but not all those equivalent by symmetry) can be blocked and in this way anisotropy can be easily induced [60]. A typical model for a metal surface in equilibrium with surfactants is shown in Figure 3.

### Complex materials

Complex materials such as those proposed in hydrogen storage also benefit from nanoparticle properties that are linked to improved atomic transport crucial for reversibility and fast kinetics of hydrogen cycling. For instance, lithium borohydride (LiBH_4_) has a high gravimetric and volumetric hydrogen density, and it was considered recently as a promising hydrogen storage material or as new superionic conductor [61–62]. The lack of the long range order in LiBH_4_ confined in 2–4 nm pores in nanoporous carbon is connected to the reduction of the barriers for the rotational motion of BH_4_^−^ anions and the high- temperature behavior even at temperatures well below the bulk *T_c_* [63]. The experimental insight to the properties of nano-confined LiBH_4_ encounters severe difficulties: Techniques such as X-ray diffraction (XRD) or neutron scattering are not always applicable to systems with reduced length-scales and those consisting of the light elements. Only the local methods, such as solid state NMR, quasielastic neutron scattering (QENS), provide indirect information about structural properties.

**Figure 4:**
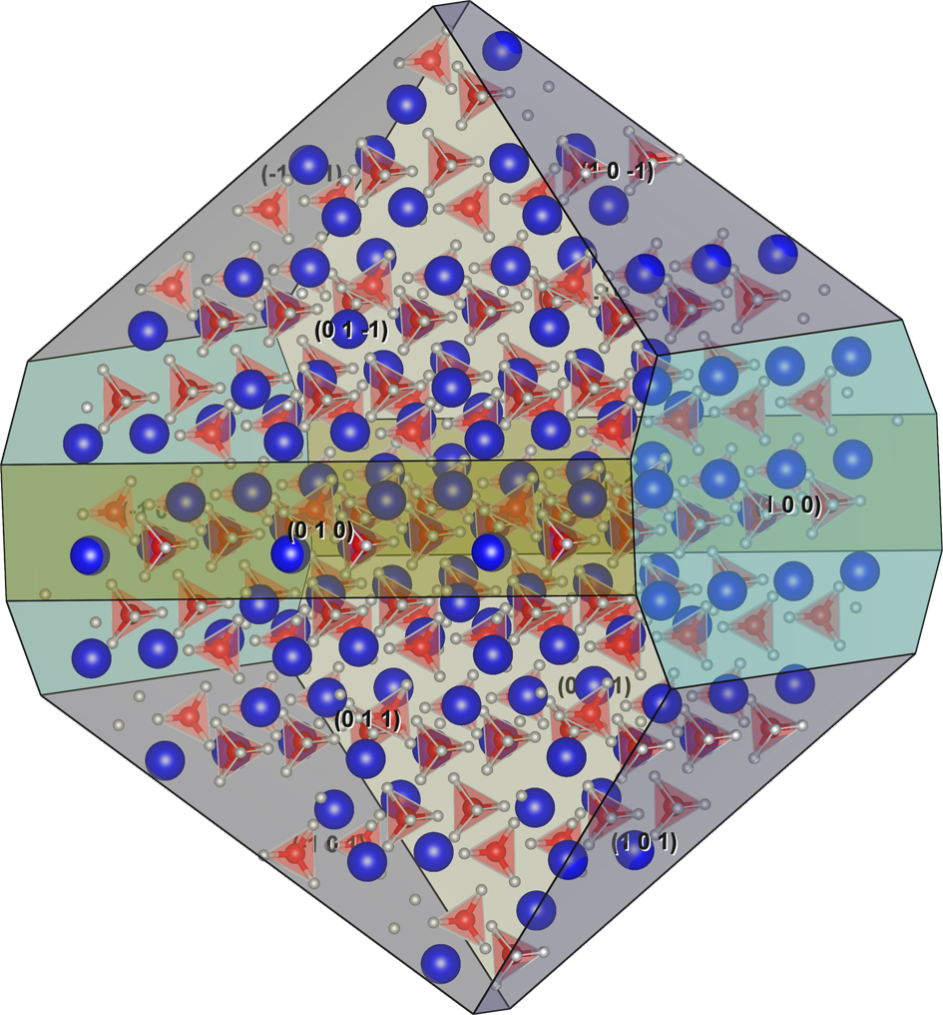
Wulff construction for the nanoparticles of LiBH_4_. The blue spheres are for lithium, red for boron and white are hydrogen atoms.

Theoretical understanding of the nanocrystalline LiBH_4_ can provide an insight into structural and dynamical properties of crystallites confined in the smallest pores with dimension below 3 nm. The Wulff construction is the starting point for these studies. The surface energy of the low-index facets of LiBH_4_ was reported and the most consistent data point out that for (100), (010), (101), (011) facets the surface energy is below 0.115 J/m^2^ [64]. The shape of the nanocrystallite is presented in Figure 4.

Contrary to the pure elements or metal oxides, for lithium borohydride the Wulff construction of the nanocrystal does not provide features that can confirm or match experimental evidence. The reason is the low surface energy of compound, fast rotational motion of anions, and large enthalpy of formation of small clusters. Due to the weak interaction with the host matrix the shape of LiBH_4_ cluster is not perturbed by the interfacial interaction. However, NMR experiments indicate a larger shielding of the boron nucleus (^11^B) in nanoconfined state, this can be explained only if the coordination of BH_4_^−^ anions is lower than three (the coordination number is four in the bulk) [65]. Such low coordination is possible only for two dimensional structures; the surface anions in the Wulff shape crystal have coordination three. However, the intensity of NMR lines suggest that the number of low coordinated atoms is larger than the number found in the Wulff construction.

## Conclusion

The Wulff construction offers a simple way to characterize nanoparticles, based on symmetry and a few parameters (ratios of surface tensions and symmetry type). Coupled to first-principles calculations for surface- and interface tensions of crystals, it proves a powerful tool that can successfully account for the nanoparticle shapes observed in experiments, including nanoparticles that interact strongly with their environment. Being a multi-scale, first-principles thermodynamics technique, it offers a parameter-free model for the key components of modern functional materials. Recent additions to the method can account for detailed atomistic structure of the nanoparticles, including coordination numbers of their atoms and density of active sites, while it can even take into account complex crystal structures and attached ligands. As such, it is expected to play a key role within theoretical materials science in the years to come.
